# A 1,000 Feet under Ground

**Published:** 1892-11

**Authors:** 


### A 1,000 FEET UNDER GROUND.

Many of our readers are unaware of the immense business involved in the underground working of a great mine. To such the following description of a descent into the great Restof Salt Mine, at York, taken from an English paper, will be full of interest.

You may imagine that you would like to go down that shaft, but let us tell you that when you once stood on the verge of that yawning hole waiting for the car to let you down, it is two to one that your courage would fail you, and you would inform the guide that you would postpone the trip until some other day. Superintendent Chapin was the one who went with us, and, of course, there was no cause for alarm, as he knows the workings of the whole plant from one end to the other. Well, we jumped into the car and waited for the signal to start, and we did not have to wait long before the gong sounded, and that was the signal to let her go. From that time until we reached the bottom nc man will ever be able to fully describe the experience.

When the signal sounds, the first thing you do is to hug your hat down on your head for keeps, and by the time you have got that act accomplished it seems as though you were going down at about the rate of a thousand miles a minute. You have seen one streak of greased lightning chase another—well, it's no comparison. It seems as though the cable has been cut, and you are just dropping down to the bottom. You can't see and the only thing to be heard is that terrible roar of the air as you rush through space. After the first two or three hundred feet there is a feeling it would be hard to explain, sort of a goneness, as it were, and you don't care much whether school keeps or not, and the changes are so different and varied that one begins to wonder what will come next.

When near the bottom the car on which you are riding begins to slow up, and then comes the most peculiar experience of all. You imagine that you are shooting upward, and you will soon be among the stars. You can imagine the sensation from going down at the rate of about 100 miles a minute to going up at about seven times that rate. Finally the car lands at the bottom of the shaft, and you breathe a sigh of relief as you step out.

Well, the first thing you do is to look for salt; it's there, all around you, above, beneath, on all sides, but it don't look much like salt near the bottom of the shaft, as lights are burned constantly and the smoke has blackened the walls. You look away to the east, through a long, dark tunnel, and you discern in the far distance some flickering lights, and you are informed that they are lights used by the workmen who are engaged in mining the salt. Your guide steps up to a man near where you land and says, "Three lights, please," and three tallow candles are handed out. It may seem a little strange that tallow candles are used in this age of kerosene, gas, and electricity, but such is the case, and they are the only lights used in the mine, and each man carries one, and they are hung up from the ceiling where the mining of salt is going on, and they are the handiest lights that can be used. They don't purchase these lights by the dozen or hundred, but by the carload.

The candles were lighted and with them in hand we followed the guide and proceeded to make a tour of the mine; we might add, a partial tour, for it would take a person something like a week to walk all over the mine territory. We followed the guide along through dark and winding pathways, until we reached a point where the workmen were busily engaged mining the salt They were not at work with picks picking it out, as might be supposed, but were breaking up the large lumps and shovelling it into the cars, the salt having been blasted out ahead of them. While some were engaged in shovelling the salt, others were drilling holes into the solid mass, making ready for a blast, machines run by compressed air being used for this purpose.

As before stated, the main tunnel runs directly east, and is nearly half a mile in length. Near the shaft two other tunnels branch off from the main tunnel, one on either side, and run parallel with it. These, we believe, are termed air shafts. From these shafts rooms branch off both north and south, and in these rooms is where the salt is mined. These rooms are nothing more nor less than short tunnels, and in time will probably be lengthened out as far as the main tunnel, or even further, as they can go miles in any direction and still be in the salt. The rooms are perhaps 20 to 30 feet wide, and from 7 to 8 feet in height. A section of salt some 30 feet in thickness is left between each room as a support to the solid mass above. A thickness of five or six feet is left above as a roof, and a substantial roof it makes, as the salt in its natutal state is almost as hard as rock. There are no other supports than the columns of salt that are left.

Of these rooms mentioned there are fifty or sixty at the present time, and the workmen are distributed about, working in several rooms at a time. There is no necessity of a foreman in each room, as the number of car-loads of salt delivered at the shaft tells the tale as to whether the men are shirking their duty or not. A railway runs through the main tunnel, and branches extend in all directions. The cars are hauled from the several rooms, by large, powerful mules, and there are some thirty of these in the mine.

There is a blacksmith shop in the mine where the tools are repaired and the mules are shod, and there is also a large stable where the mules are sheltered during the night. Of course, they would be well sheltered in the mine, any way, but if allowed to roam about they could find nothing to eat but salt and the railroad track, and the average mule cannot exist on a diet of this kind. This stable is far ahead of the ordinary stable about the country, and there is every convenience and luxury for his mule-ship. The stables are some 40 or 50 feet in length, and 20 or 30 feet wide, with wood floor and wooden stalls and mangers. This is the only combustible substance there is about the mine, and there are no exposed lights any where about it. Directly in the rear of the stables is what is known as the barnyard. This is a large room cut in the solid salt, and here the mules are turned out for recuperation.

One may imagine that a salt mine is a bad place to work, but aside from the fact that it is a little dismal, there are no bad features about it. Unlike a coal mine, it is clean, and there is almost an even temperature the year around, and ranging from 58° to 60°, winter and summer. The ventilation is perfect, and the system for supplying fresh air is not excelled by any mine in the world. In some of the passageways the air rushed through with such velocity as to extinguish the lights.

The experience in going up the shaft is somewhat different from that while going down. The signal is given from below after you have been safely stationed in the car, and away she goes, your hat sinks down firmly on your head, and your clothing seems to sit right down tight where it belongs. A person who is a little weak in the knees would also have a tendency to sit right down tight on the bottom of the car. The roar of the wind as you hustle up toward daylight is about all that can be heard. When near the top the speed is lessened, and it is then that one imagines that he is going down again at the rate of about 1000 miles a minute, but finally the daylight begins to peep down at you and you are landed safely on top, only a few seconds having elapsed since you walked upon the car below.

